# A novel MRI feature, the cut green pepper sign, can help differentiate a suprasellar pilocytic astrocytoma from an adamantinomatous craniopharyngioma

**DOI:** 10.1186/s12880-023-01132-0

**Published:** 2023-11-20

**Authors:** Shumin Xu, Wanqun Yang, Yi Luo, Xiaoyu Wang, Yaowen Li, Xianlei Meng, Yuze Zhang, Hongwu Zeng, Biao Huang

**Affiliations:** 1 Department of Radiology, Shenzhen Children’s Hospital, Shenzhen, 518000 China; 2 Department of Radiology, Guangdong Provincial Key Laboratory of Artificial Intelligence in Medical Image Analysis and Application, Guangdong Provincial People’s Hospital, Guangdong Academy of Medical Sciences, Guangzhou, 514000 China

**Keywords:** Cut green pepper sign, Pilocytic astrocytoma, Adamantinomatous craniopharyngioma, Magnetic resonance imaging, Differential diagnosis

## Abstract

**Objective:**

There are no specific magnetic resonance imaging (MRI) features that distinguish pilocytic astrocytoma (PA) from adamantinomatous craniopharyngioma (ACP). In this study we compared the frequency of a novel enhancement characteristic on MRI (called the cut green pepper sign) in PA and ACP.

**Methods:**

Consecutive patients with PA (n = 24) and ACP (n = 36) in the suprasellar region were included in the analysis. The cut green pepper sign was evaluated on post-contrast T1WI images independently by 2 neuroradiologists who were unaware of the pathologic diagnosis. The frequency of cut green pepper sign in PA and ACP was compared with Fisher’s exact test.

**Results:**

The cut green pepper sign was identified in 50% (12/24) of patients with PA, and 5.6% (2/36) with ACP. The sensitivity, specificity, positive predictive value (PPV), and negative predictive value (NPV) of the cut green pepper sign for diagnosing PA were 50%, 94.4%, 85.7% and 73.9%, respectively. There was a statistically significant difference in the age of patients with PA with and without the cut green pepper sign (12.3 ± 9.2 years vs. 5.5 ± 4.4 years, *p* = 0.035).

**Conclusion:**

The novel cut green pepper sign can help distinguish suprasellar PA from ACP on MRI.

**Supplementary Information:**

The online version contains supplementary material available at 10.1186/s12880-023-01132-0.

## Introduction

Pilocytic astrocytoma (PA) is the most common primary brain tumor found in children and adolescents (up to 19 years old), and accounts for about 15% of tumors. The incidence of PA decreases with increasing age [1]. PA is typically classified as a grade 1 astrocytic tumor, according to the 5th edition of the 2021 World Health Organization (WHO) Classification of Tumors in the Central Nervous System (CNS) [2]. The prognosis of PA is excellent if complete surgical resection is achieved, with a 5-year survival rate of 94% [1, 3]. While PA can occur throughout the neuro-axis, the majority (67%) are found in the cerebellum and suprasellar region [4].

A PA in the suprasellar region has similar clinical presentations and radiographic features as adamantinomatous craniopharyngioma (ACP) [5–7]. Radiographically, both tumors typically present as a cystic-solid mass with heterogeneous enhancement. Calcification is more common in ACP; however, a proportion of ACP have little or no calcifications, and particularly in adults a substantial number of ACP do not have calcifications [8–10]. Calcifications can be present in PA, especially lesions arising from the hypothalamic or optic nerve [11]. Additionally, ACP may present with edema along the optic tracts (the moustache sign), and thus may be confused with PA arising from the optic tract [12]. The treatment and prognosis for PA and ACP are very different [9, 13, 14]. An early and accurate diagnosis is essential for determining a surgical strategy and effective treatment. Thus, differentiating between PA and ACP in the suprasellar region is extemely important.

PA is a slow-growing tumor, which is usually accompanied by tumor degenerative atypia. Recently, we have observed an irregular rim of peripheral enhancement with indentations on the surface of PA on MRI; an imaging sign we have termed the “cut green pepper sign” due to its visual appearance. We hypothesized that the cut green pepper sign is a characteristic MRI feature of PA. Thus, the purpose of this study was to determine if the cut green pepper sign is useful for distinguishing PA and ACP in the suprasellar region on MRI.

## Materials and methods

### Study Design

The records of consecutive patients with PA or ACP in the suprasellar region seen at 2 institutions from January 2013 to December 2021 were retrospectively reviewed. The inclusion criteria were: (1) Histopathological confirmation of PA or ACP based on the criteria in the 5th edition of the WHO Classification of Tumors of the CNS; (2) Complete medical record; (3) Had not undergone radiotherapy or surgical treatment prior to the first MRI scan; and (4) MRI sequences including, but not limited to, T2WI, FLAIR (fast fluid-attenuated inversion recovery), T1WI (pre- and post-contrast).

### MRI acquisition

MRI was performed before surgical resection, with a scanner with a field strength of 1.5T or 3.0T. Although there were some differences in MRI protocols between the 2 hospitals, sequences of interest were the same at the 2 hospitals. The key sequences were axial fast spin-echo T2WI, axial FLAIR, axial spin-echo T1WI, and contrast-enhanced (CE) T1WI. Postcontrast T1WI, axial, sagittal, and coronal plane images were acquired after intravenous injection of gadopentetate dimeglumine (Gd-DTPA, Magnevist; Bayer Schering Pharma, Berlin, Germany) at a dose of 0.1 mmol/kg.

### Definition of the cut green pepper sign

**Fig. 1:**
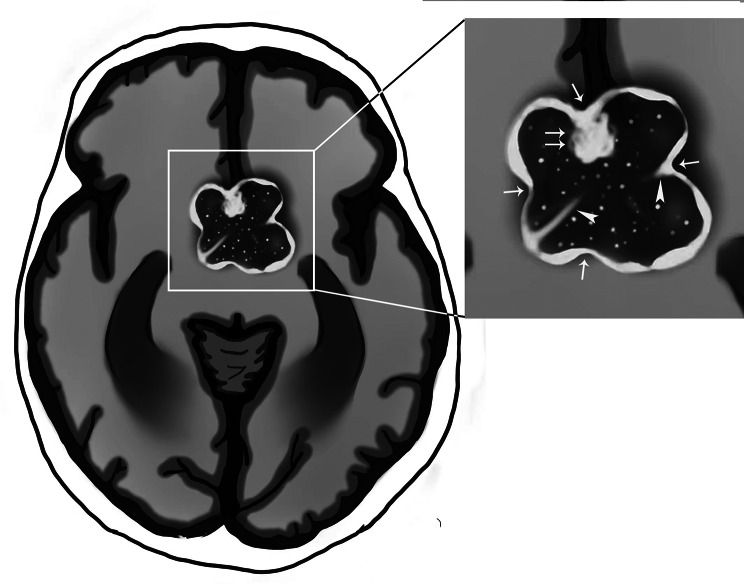
Illustration of the cut green pepper sign. Suprasellar lesion with irregular rim enhancement that resembles a cut section of a green pepper. There are crests (arrowhead) and a mural nodule (double arrows) on the inner edge. Several indentations (arrow) are similar to the surface of a green pepper. Discrete punctate and patchy enhancement inside the tumor resembles scattered green pepper seeds

The cut green pepper sign was defined as irregular peripheral rim enhancement, or ground-glass-like appearance enhancement, with or without a strongly enhancing nodule in the lesion. The appearance is similar to that of the flesh of a cut green pepper (Fig. 1). Also, on CE-T1WI images there were at least 2 indentations on the surface of the lesion similar to the surface of a green pepper, 1 or more crests on the inner edge, and discrete point-like enhancement inside the tumor resembling the appearance of scattered green pepper seeds. Definition of the cut green pepper sign required the aforementioned imaging characteristics present on at least 1 slice in the transverse, coronal, or sagittal planes on CE-T1WI scans.

### MRI review

All patient clinic data and images were mixed and numbered randomly, and then independently analyzed in a blinded fashion separately by 2 neuroradiologists. The reviewers examined images for the presence or absence of the cut green pepper sign based on post-Gd-DTPA gadolinium T1WI. If they did not reach consensus, a senior neuroradiologist was consulted to make the final decision. For PA, the maximum size of the enhancing lesion was measured in millimeters on CE-T1WI.

### Clinical data

Clinical and demographic data, including age, sex, and clinical signs and symptoms were extracted from electronic medical records.

### Statistical analysis

SPSS 22.0 software was applied for statistical analysis. Continuous data (age and maximum tumor diameter) were presented as mean ± standard deviation; categorical data (gender and MRI features) as count and percentage. The independent sample t-test or Mann-Whitney *U* test was used to compare the difference of continuous data between the two groups, and the Chi-square test or Fisher’s exact test was used to compare the difference of categorical variables between the two groups. Pearson’s chi-squared test was used to compare the frequency of sexes between the two groups. The prevalence of the cut green pepper sign in patients with PA and ACP was compared using Fisher’s exact test. The sensitivity and specificity of the cut green pepper sign for diagnosing PA was calculated using receiver operating characteristic (ROC) curve analysis. The area under the curve (AUC), confidence interval, optimal critical value and other evaluation indexes were obtained; two-sided test was used. Values of *p* < 0.05 were considered to indicate a statistically significant difference.

## Results

### Patient demographic and clinical characteristics

A total of 63 patients with a PA or ACP were identified in the medical records. Three patients were excluded due to poor imaging quality; thus 24 patients with PA and 36 with ACP were included in the analysis. The main clinical features and imaging findings of the 60 patients are summarized in Table 1.

**Table 1:** Demographic data and imaging features

	PA (n = 24)	ACP (n = 36)	*p* value
**Age(years)** ^a^			
**mean**	8.9 ± 7.8	17.9 ± 19.7	0.171
**Gender** ^b^			
**M/F**	13/11	19/17	0.916
**Max diameter (mm)** ^c^	44.3 ± 14.6	32.1 ± 12.7	0.001^*^
**cut green pepper sign** ^d^			
**yes/no**	12/12	2/34	< 0.001^*^

^*a*^ *Mann–Whitney U-test;* ^*b*^ *Chi-square test;* ^*c*^ *independent samples t-test;* ^*d*^ *Fisher’s exact test.* ^*^ *p < 0.05 indicated statistical significance*

**PA**: *pilocytic astrocytoma*

**ACP**: *adamantinomatous craniopharyngioma*

The PA group was comprised of 13 males and 11 females, with a mean age of 8.9 ± 7.8 years (range, 4 months to 31 years). The ACP group was comprised of 19 males and 17 females with a mean age of 17.9 ± 19.7 years (range, 6 months to 64 years). There was no significant difference between the PA group and ACP group with respect to sex (*p* = 0.916) and age (*p* = 0.171). The main symptoms at the time of diagnosis were headache, vomiting, and other symptoms of elevated intracranial pressure, visual disturbances, and endocrine disorders.

### Cut green pepper sign in PA

**Fig. 2:**
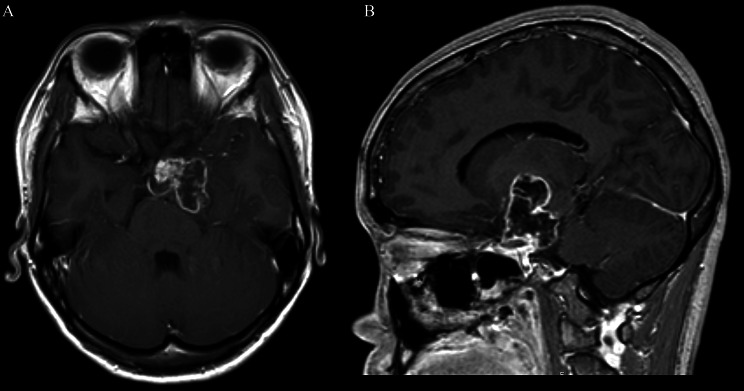
A 21-year-old patient with a suprasellar PA. Axial (**A**) and sagittal (**B**) postcontrast T1-weighted images show an example of the cut green pepper sign. The axial image shows several indentations on the surface of the lesion, and a marked enhancing mural nodule. The sagittal image shows discrete point-like enhancement inside the tumor, that resembles scattered green pepper seeds

**Fig. 3:**
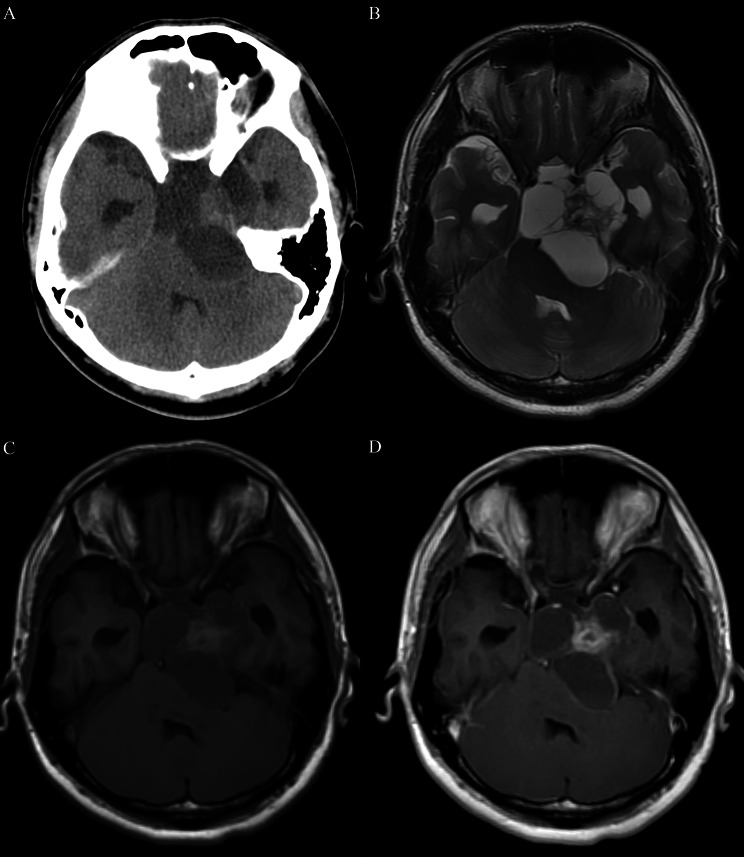
A 23-year-old patient with a suprasellar PA. (**A**) Axial CT image shows a cystic-solid heterogeneous suprasellar mass. (**B**) Axial T2-weighted image demonstrates predominant hyperintensity of the mass, with slight hypointensity of the nodule. (**C**) Axial T1-weighted image reveals hypointensity of the mass, with slight hyperintensity of the nodule. (**D**) Axial postcontrast T1-weighted image shows irregular rim enhancement with a mural nodule and multiple crests connecting the indentations on the surface, which is similar to the appearance of the flesh of a cut green pepper

**Fig. 4:**
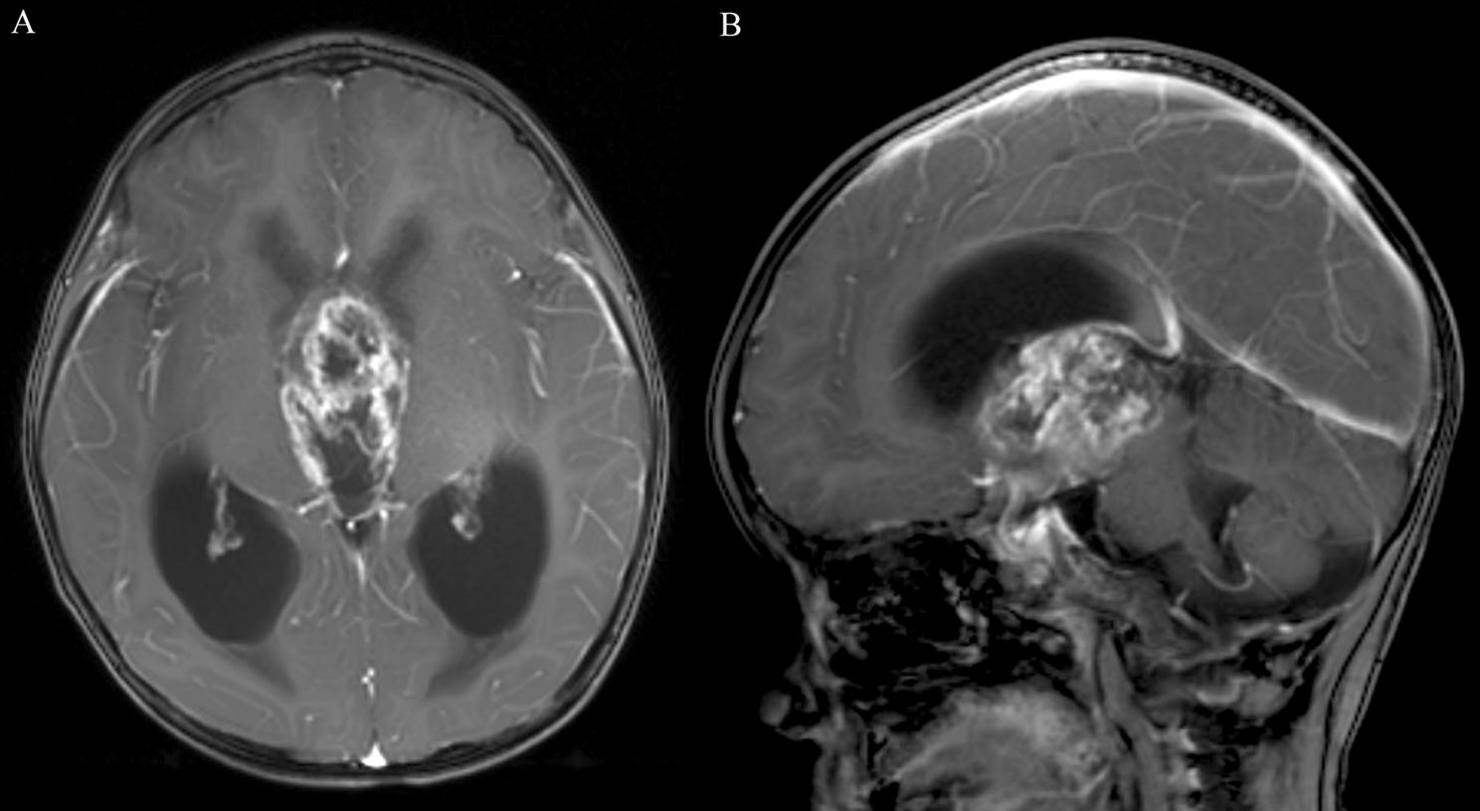
A 4-year-old patient with a suprasellar PA. Axial (**A**) and sagittal (**B**) postcontrast T1-weighted images show a peripherally ground-glass-like appearance enhancing lesion with crests on the inner edge. The lesion resembles the flesh of a longitudinal section of a cut green pepper with multiple indentations on the surface, and discrete point-like enhancement inside the tumor

The cut green pepper sign was present in 50% (12/24) of patients with PA. There were 2 types of imaging pattern; cystic-solid and non-cystic-solid. In the cystic-solid group, 12 of 16 patients had the cut green pepper sign, including 4 adults and 8 children. For the 4 adult cases, 3 cases were misdiagnosed as ACP, one of them presenting as an irregular, peripherally enhancing suprasellar lesion with discrete point-like enhancement inside the tumor (Fig. 2); the other one was misdiagnosed as germ cell tumor, presenting as a heterogeneous enhancing mass with an intense enhancing mural nodule (Fig. 3). For one case of children, the cut green pepper sign was seen in both axial and sagittal images, showing peripheral ground-glass-like appearance enhancement and crests on the inner edge, resembling the longitudinal section of a cut green pepper (Fig. 4).

The cut green pepper sign was absent in the 8 cases classified in non-cystic-solid.

### Cut green pepper sign in ACP

**Fig. 5:**
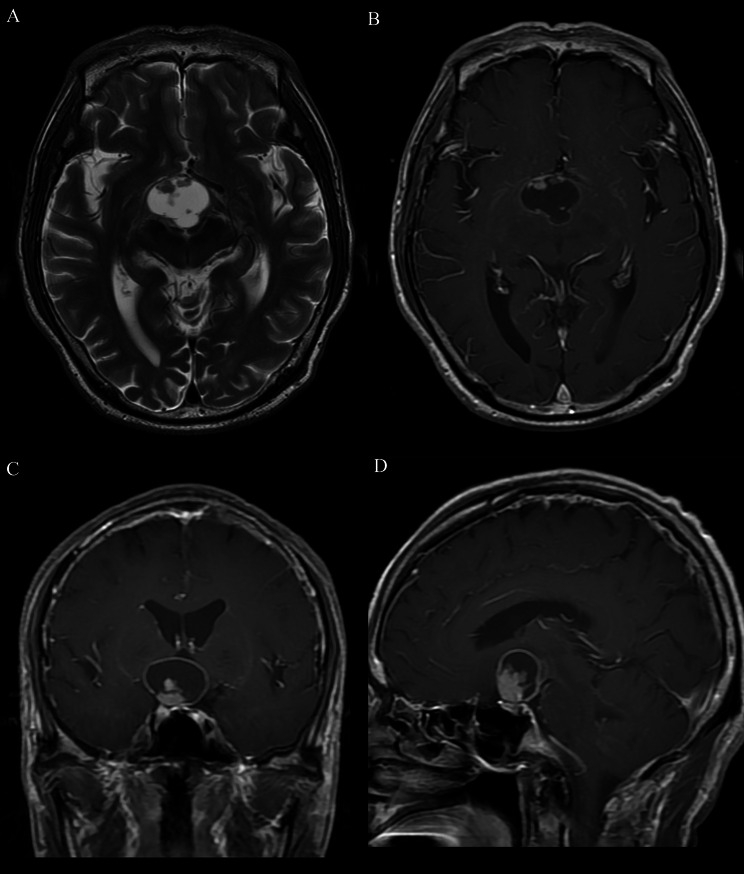
A 63-year-old patient with a suprasellar ACP. (**A**) Axial T2-weighted image demonstrates a cystic-solid mass. (**B**) Axial postcontrast T1-weighted, (**C**) coronal postcontrast T1-weighted, and (**D**) sagittal postcontrast T1-weighted images illustrate ring enhancement with a marked enhancing mural nodule. Unlike the cut green pepper sign, the inner wall of the tumor is smooth, there is no crest, and there is no green pepper seed-like enhancement inside the tumor

The cut green pepper sign was present in 5.6% (2/36) of patients with ACP. The remaining 34 cases absence of cut green pepper sign. One case absence of cut green pepper sign in ACP, presenting as a ring enhancing lesion with two indentations on the surface and an intense enhancing mural nodule. Unlike cut green pepper sign, the inner wall is regular, and presents without discrete point-like enhancement inside the tumor (Fig. 5).

### Diagnostic value of the cut green pepper sign for PA

**Fig. 6:**
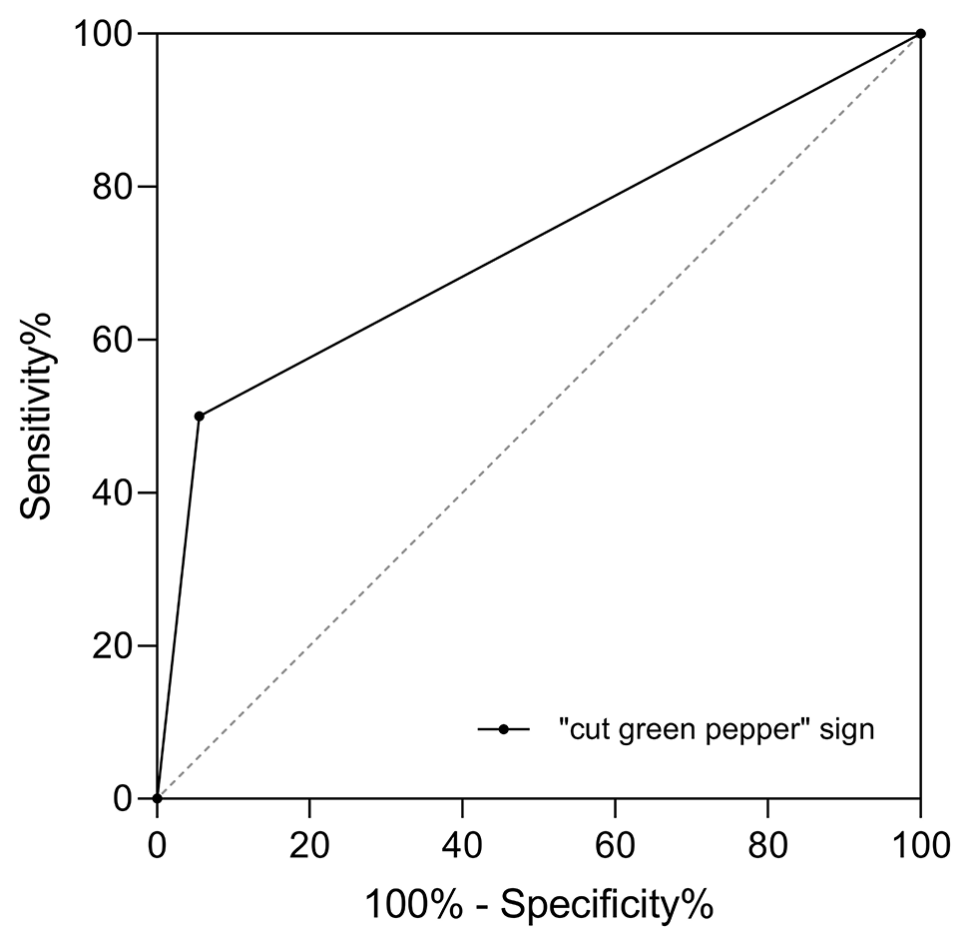
Receiver operating characteristic curves of the cut green pepper sign for identifying PA. The AUC was 0.72 (95% CI: 58–86%)

The presence of the cut green pepper sign exhibited a sensitivity of 50% (12/24), specificity of 94% (34/36), PPV of 86% (12/14), and NPV of 74% (34/46) for diagnosis of PA. The area under the ROC curve was 0.72 (95% confidence interval (CI): 58–86%) (Fig. 6).

### Diagnostic value of the cut green pepper sign for cystic-solid PA

The presence of the cut green pepper sign exhibited a sensitivity of 75% (12/16), specificity of 94% (34/36), PPV of 86% (12/14), and NPV of 90% (34/38) for diagnosis of cystic-solid PA. The AUC was 0.85 (95% CI: 71–98%).

### Association of maximum size and age of patient with PA with the presence or absence of the cut green pepper sign

In PA group, there was a statistically significant difference in age between patients with and without the cut green pepper sign (*p* = 0.035). The mean age of PA patients with the cut green pepper sign were 12.3 ± 9.2 years, and that of patients without the cut green pepper sign were 5.5 ± 4.4 years. The maximum diameter of the PA in patients with and without the cut green pepper sign was not significantly different (45.3 ± 15.4 mm vs. 43.4 ± 14.3 mm, *p* = 0.766).

## Discussion

This study demonstrated that the cut green pepper sign, an irregular rim of peripheral enhancement with indentations on the surface, is highly specific for a suprasellar PA compared to an ACP. Sensitivity, specificity, PPV, and NPV of the cut green pepper sign for diagnosing PA were 50%, 94%, 86%, and 74%, respectively. The cut green pepper sign may be associated with indolent, and occasionally degenerative biologic behavior of a PA. The cut green pepper sign can help to preoperatively differentiate a suprasellar PA from an ACP.

The variations in imaging patterns of a PA make a correct diagnosis challenging [15]. There are 4 predominant imaging patterns of a PA: (1) A mass with a marked enhancement of a mural nodule and enhancing cyst wall (46%); (2) A mass with a marked enhancement of a mural nodule and non-enhancing cyst (21%); (3) A predominately solid mass with minimal to no cyst-like component (17%); and (4) A necrotic mass without central enhancement (16%) [16]. Suprasellar PA and ACP are relatively common in children, and traditional imaging techniques are of limited value in distinguishing between the 2 lesions [6, 7, 12]. Eggshell-like calcifications and hyperintense cystic components on T1-weighted images are more common in ACP; however, their sensitivity is low [17, 18]. Various functional MRI techniques have been used to differentiate the 2 lesions. MR spectroscopy may become a useful tool in differentiating ACP from PA by depicting prominent peaks of lipids and cholesterol. Diffusion Tensor Imaging (DTI) has also been proven useful in differentiating the PA from other suprasellar tumors [19, 20]. However, these methods have limited value and low sensitivity, increase the cost and duration of examinations for patients, and are difficult to obtain in some hospitals.

In this study, the imaging pattern of PA enhancement resembling a cut green pepper was highly specific for diagnosis of PA compared with ACP. To the best of our knowledge, this study was the first to describe and apply a novel MRI finding, the cut green pepper sign, to distinguish PA from ACP in suprasellar region. The use of this feature may aid the diagnosis of PA.

The treatments and prognosis for PA and ACP are different [21–24]. As the site of origin and different biologic behavior of PA, the treatment options are diverse. Management strategies range from conservative monitoring, to biopsy, subtotal resection, total resection, radiotherapy and chemotherapy [25, 26]. For ACP, traditional treatment options include surgery and radiotherapy. However, aggressive neurosurgical intervention can severely reduce quality of life due to high recurrence rates and long-term complications. Recent development of targeted and intra-cystic therapies via an indwelling catheter to aspirate cystic fluid or to administer medications have been shown to be effective in improving long-term control of tumor volume and reducing morbidity [9, 27].Obviously, accurate preoperative identification of PA versus ACP is essential to guide preoperative decision making, for patient and family counseling, and to assess clinical prognosis.

Although the exact mechanism that results in the cut green pepper sign is unclear, MRI finding of cut green pepper sign may be associated with the special histopathological. The indolent and occasionally spontaneous degenerative biologic behavior of PA results in unique histopathological changes. Histologically, PA exhibits a biphasic pattern consisting of loose-textured multipolar cells associated with microcysts and eosinophilic granular bodies, and compacted bipolar cells associated with Rosenthal fibers [28]. Local shrinkage may occur as the lesion degenerates, and the indentations and crests of a PA may be related to shrinkage of the loose-textured tumor parenchyma [29]. Degenerative changes are more often observed in older patients with PA. In this study, the mean age of PA patients with the cut green pepper sign was older than that of those without the sign. In this study, 4 adult patients all presented with the cut green pepper sign. The main manifestations of degeneration are vascular hyaline degeneration and cystic degeneration. Vascular hyaline degeneration may lead to disturbance of the blood-brain barrier, resulting in significant enhancement on MRI [30]. The cystic component of a PA is rich in vascular growth factors, which stimulate vascular proliferation. Glomeruloid vasculature is seen within the tumor and cyst walls, causing a narrow band of contrast enhancement at the circumference of some cysts [4]. In present study, PA with the cut green pepper sign exhibited obvious vascular degeneration and glomeruloid vasculature on histopathological examination. We speculate that peripheral enhancement and the green pepper seed-like enhancement of tumor parenchyma are related to this.

This study had several limitations. First, because the overall incidence of PA and ACP is low in brain tumors. the number of patients in the study was relatively small. However, the analysis did find statistical differences between the 2 groups. Second, only patients who underwent surgical resection were included in this study; patients with smaller lesions that did not cause symptoms and patients with lesions not suitable for surgical resection were not included in the study. Finally, as a retrospective study, certain potential selection biases could not be excluded.

## Conclusion

In conclusion, the cut green pepper sign, an irregular rim of peripheral enhancement of a PA, is useful for differentiating a PA from ACP in the suprasellar region. A suprasellar PA can be difficult to accurately diagnose because they can have clinical and radiographic presentations very similar to an ACP. Identification of the cut green pepper sign on MRI can help distinguish a suprasellar PA from an ACP.

### Electronic supplementary material

Below is the link to the electronic supplementary material.

Supplementary Material 1

## Data Availability

The datasets used and/or analysed during the current study available from the corresponding author on reasonable request.
