# Supplementary figures and images for: A novel MRI feature, the cut green pepper sign, can help differentiate a suprasellar pilocytic astrocytoma from an adamantinomatous craniopharyngioma
Source: BMC Med Imaging. 2023 Nov 20;23:191. doi: 10.1186/s12880-023-01132-0 (PMC10662486; doi:10.1186/s12880-023-01132-0)

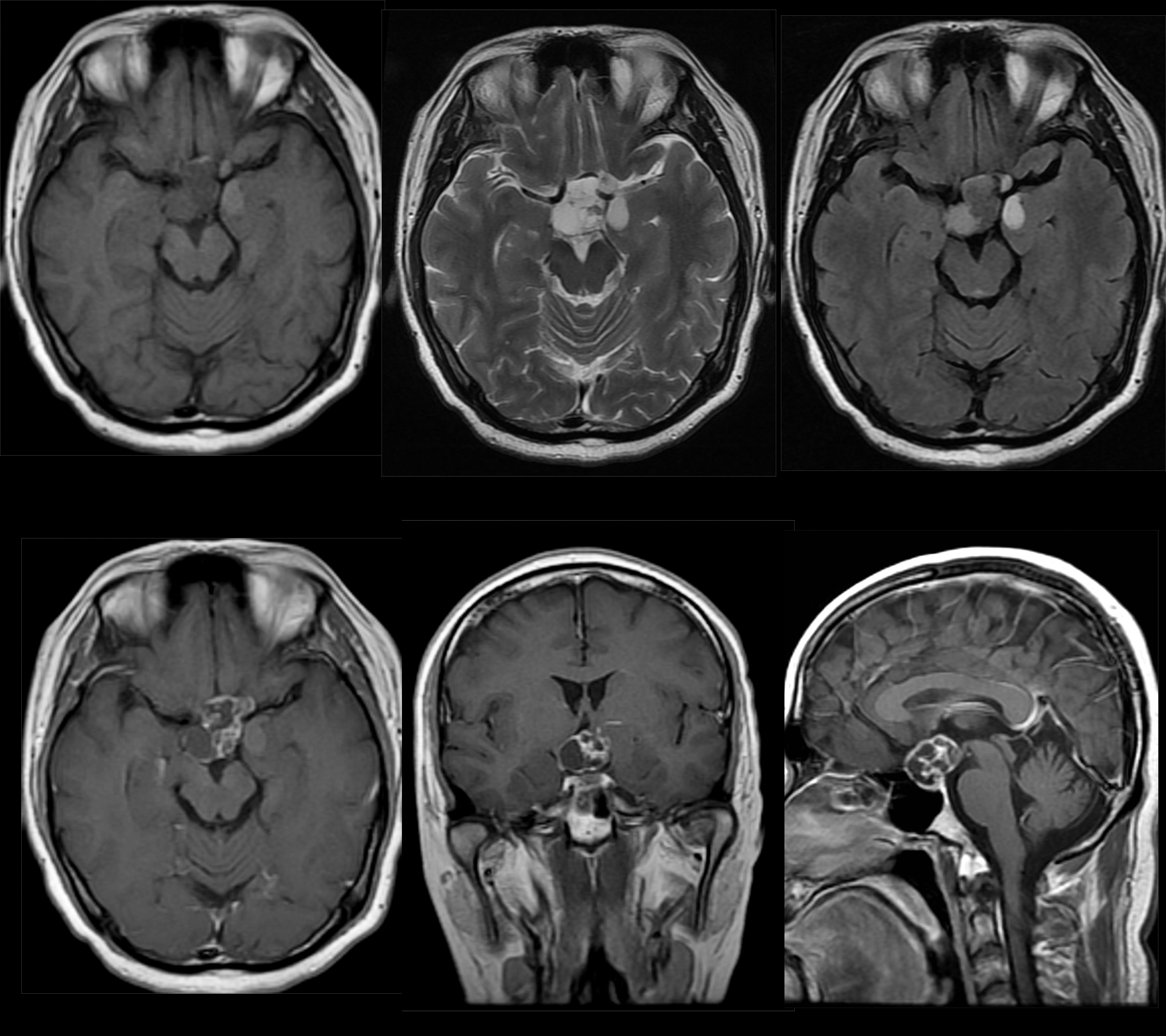

Supplement: Supplementary file 1 — Supplementary Material 1 [file 12880_2023_1132_MOESM1_ESM.jpg]
